# Mechanical Stress Promotes Cisplatin-Induced Hepatocellular Carcinoma Cell Death

**DOI:** 10.1155/2015/430569

**Published:** 2015-01-22

**Authors:** Laila Ziko, Sandra Riad, Momen Amer, Radovan Zdero, Habiba Bougherara, Asma Amleh

**Affiliations:** ^1^Biotechnology Program, School of Sciences and Engineering, The American University in Cairo, AUC Avenue, New Cairo 11835, Egypt; ^2^Department of Biology, School of Sciences and Engineering, The American University in Cairo, AUC Avenue, New Cairo 11835, Egypt; ^3^Department of Mechanical and Industrial Engineering, Ryerson University, 350 Victoria Street, Toronto, ON, Canada M5B 2K3; ^4^Martin Orthopaedic Biomechanics Lab, St. Michael's Hospital, 209 Victoria Street, Toronto, ON, Canada M5B 1W8; ^5^Department of Mechanical and Industrial Engineering, University of Toronto, Toronto, ON, Canada M5S 3G8

## Abstract

Cisplatin (CisPt) is a commonly used platinum-based chemotherapeutic agent. Its efficacy is limited due to drug resistance and multiple side effects, thereby warranting a new approach to improving the pharmacological effect of CisPt. A newly developed mathematical hypothesis suggested that mechanical loading, when coupled with a chemotherapeutic drug such as CisPt and immune cells, would boost tumor cell death. The current study investigated the aforementioned mathematical hypothesis by exposing human hepatocellular liver carcinoma (HepG2) cells to CisPt, peripheral blood mononuclear cells, and mechanical stress individually and in combination. HepG2 cells were also treated with a mixture of CisPt and carnosine with and without mechanical stress to examine one possible mechanism employed by mechanical stress to enhance CisPt effects. Carnosine is a dipeptide that reportedly sequesters platinum-based drugs away from their pharmacological target-site. Mechanical stress was achieved using an orbital shaker that produced 300 rpm with a horizontal circular motion. Our results demonstrated that mechanical stress promoted CisPt-induced death of HepG2 cells (~35% more cell death). Moreover, results showed that CisPt-induced death was compromised when CisPt was left to mix with carnosine 24 hours preceding treatment. Mechanical stress, however, ameliorated cell death (20% more cell death).

## 1. Introduction

Chemotherapy continues to be a common method for treating cancer, and most treatment regimens demand high dosing of chemotherapeutic agents. Among the most widely used chemotherapeutics are platinum- (Pt-) based drugs such as cisplatin (CisPt) and oxaliplatin (OxPt). Two main limitations of these agents, like other chemotherapeutic drugs, are multiple reported side effects [1, 2] and limited drug efficacy due to the development of drug resistance. The side effects and drug resistance are both believed to be consequences of the chemotherapy drugs' mechanism of action, which is mainly directed at halting cell division by damaging DNA. Side effects arise because the Pt-based drug effect is not restricted to cancer cells; it influences the normal cells that continuously proliferate as well [2–6]. The current study focuses on increasing the effect of CisPt at a low dose thereby enabling a lower dose to be administered, resulting in fewer side effects caused by the fact that CisPt is inherently not specific to cancer cells. Furthermore, the increase in the cancer cell killing effect, in essence, would attain increased CisPt efficacy.

Current alternative approaches focus on avoiding the cytotoxic effects of anticancer drugs on noncancerous cells and eliminate cancer cells more specifically. Such approaches mainly introduce novel chemotherapeutics that kill only cancer cells while leaving normal cells unharmed [7, 8]. Current treatments essentially target molecules that contribute to the hallmarks of cancer [1], including newly introduced unconventional strategies which are mainly used as complementary treatments [9]. For instance, the US Food and Drug Administration has recently approved tumor treating fields (TTF), which are low current electric fields that selectively destroy cancer cells with fewer side effects [9].

In the present study, a new multistress factor approach to killing cancer cells is investigated. One theoretical hypothesis suggested that adding mechanical stress to anticancer drugs in the presence of healthy (noncancerous) immune cells could kill more cancer cells [10]. This suggestion was presented in a mathematical model which predicted that more cancer cells would be killed if biochemical reactions were stimulated with a mechanical force [10]. Both* in vivo* and* in vitro* studies have shown that cells respond to mechanical stress by activating protective genes known as heat shock proteins, such as HSP70 [11, 12]. Besides mechanical stress, it was also found that other forms of stress trigger upregulation of heat shock proteins [12]. The protective role of these proteins depends on the nature and duration of the applied stress. As stress levels and/or durations rise, the protective capability of the cell is challenged and thus the cell death cascade is switched on. In programmed cell death (apoptosis), levels of the proapoptotic molecule BAX become elevated [13]. Activated BAX, if not dampened by antiapoptotic molecules such as XIAP, will mediate the activation of Caspase-3 [14], which facilitates apoptotic cleavage of PARP-1. However, no study has experimentally verified* in vitro* whether or not mechanical stress applied with anticancer drugs in the presence of immune cells increases cancer cell death.

As per the previously mentioned mathematical model, when mechanical stress is coupled with an anticancer drug in the presence of a healthy cell that has an anticancer effect, the cancer cell survival would decrease more than if one of the previous factors were to be used alone [10]. Peripheral blood mononuclear cells (PBMCs) were used as the third factor to study whether the cancer cell survival would be affected. PBMCs comprise the immune cells present in the blood, including natural killer cells and cytotoxic T cells, that inherently have cytotoxic activity against cancer cells and hence kill a percentage of HepG2 cells when cocultured with them [15]. PBMCs are also used frequently to test compounds for their potential ability to increase the antitumor activity of the immune cells [15].

In order to decipher the specific cellular mechanism of action that is employed by mechanical stress in its effect on CisPt and eventually its cytotoxic effect, molecules that bind to CisPt in the cytoplasm are of considerable importance. CisPt resistance occurs because of the fact that not all CisPt molecules reach the nucleus where they could cause cellular death; they may instead bind to endogenous molecules present in the cytoplasm [16]. Among the endogenous molecules that have affinity for platinum-based drugs are glutathione and carnosine [16–18]. The challenge of the limited efficacy of Pt-based drugs is accentuated by the fact that while less than 10% of the uptaken drug is delivered to the nucleus, the majority of the Pt-based drug remains sequestered in the cytoplasm [16, 17]. Recently, we have demonstrated that OxPt complexes with carnosine, a cytosolic dipeptide ligand thus keeping OxPt away from the nucleus, its pharmacological target site [18]. Carnosine was also reported as having antioxidant effects on lymphocytes and protective effects against cellular DNA damage [19]. One hypothesis that might explain the mechanism of action of mechanical stress is that it might stimulate the release of CisPt from cytoplasmic molecules, for example, carnosine, hence freeing CisPt molecules that could move to the nucleus and cause cell death.

Therefore, the two main aims of this study were to first determine experimentally whether mechanical stress causes higher cancer cell cytotoxicity when added to immune cells (PBMCs) with or without the anticancer drug CisPt as compared to any of the tested factors alone. The second objective was to study one possible mechanism of action by which mechanical stress may function in increasing CisPt cytotoxic effect, the hypothesis being that mechanical stress reduces CisPt affinity towards complexation with carnosine and thus enhances the CisPt cytotoxic effect.

## 2. Materials and Methods

### 2.1. Cell Culture

A human hepatocellular liver carcinoma (HepG2) cell line was used as an* in vitro* model of liver cancer, because HepG2 cells show similar cell morphology as hepatocytes [20] and are commonly used to test immunomodulatory effects of molecules on cancer cells [15]. The HepG2 cell line was maintained in complete media composed of RPMI 1640 (Lonza, USA) supplemented with 10% heat-inactivated fetal bovine serum (FBS) (Lonza, USA) and 5% penicillin-streptomycin (Lonza, USA), incubated in 5% CO_2_ at 37°C. For MTT assays, cells were seeded at 2 × 10^4^ cells/well in 96-well plates (Greiner Bio-One, Germany). For RNA and protein analysis experiments, cells were seeded at 3 × 10^5^ cells/well in 6-well culture plates (Greiner Bio-One, Germany). After seeding, HepG2 cells were cultured overnight prior to stimulation. For morphological changes and cell death features, cells were examined using an inverted microscope (Olympus 1X70, USA). The viable cell count was determined by trypan blue staining using a hemocytometer (Hausser Scientific, USA). HepG2 cells and PBMCs were counted before seeding, in order to fix the count and ratio of PBMCs: HepG2 cells.

### 2.2. Experimental Design

Combinations of mechanical stress (rotational shear force), CisPt, and/or PBMCs were applied to HepG2 cells. On each of 3 different days, a fresh batch of 8 to 16 samples (i.e., wells) was prepared for each of the following 8 groups: control, CisPt alone, PBMCs alone, CisPt + PBMCs, mechanical stress alone, mechanical stress + CisPt, mechanical stress + PBMCs, and mechanical stress + CisPt + PBMCs. Thus, over the course of the 3 days, a total of 24 to 48 wells were used per test group. In another set of experiments, the combination of mechanical stress, CisPt, and/or carnosine was applied to HepG2 cancer cells. Similar to the previous set of experiments, a fresh batch of 8 samples (i.e., wells) was prepared for each of the following 14 groups: control, water, saline, water + saline, carnosine alone, CisPt alone, CisPt + carnosine, mechanical stress alone, mechanical stress + water, mechanical stress + saline, mechanical stress + water + saline, mechanical stress + carnosine, mechanical stress + CisPt, and mechanical stress + CisPt + carnosine. Different ratios of carnosine to CisPt (1 : 1, 1.5 : 1, 2 : 1, 2.5 : 1, and 3 : 1) were also tested. Although cell death can occur by multiple mechanisms (e.g., apoptosis, autophagic death, necrosis, etc.), the present study examined the proapoptotic molecules BAX and Caspase-3, as well as XIAP, an antiapoptotic molecule, and HSP70, a stress protein, at their RNA levels and the apoptotic cleavage of PARP-1 at the protein level [11].

### 2.3. Cisplatin

CisPt (*cis*-diammineplatinum(II)dichloride) powder was obtained (Sigma-Aldrich, USA) and dissolved in sterile-filtered 0.9% NaCl. A 1 mg/mL stock was prepared and stored at 4°C in an amber bottle. For preliminary testing, the stock solution was further diluted with a complete medium at concentrations of 0.5 to 32 *μ*g/mL in which cells were incubated for 24, 48, and 72 hours [21]. Thereafter, a final concentration of 2 *μ*g/mL was chosen to assess the effect of mechanical stress on the efficacy of CisPt. 2 *μ*g/mL was specifically chosen because, at that concentration, CisPt resulted in a low cell death percentage (~20%) which would allow the relative influence of PBMCs and/or mechanical stress to be determined during experimentation. To assess the effect of mechanical stress on CisPt binding affinity and complexation with carnosine, a final concentration of 8 *μ*g/mL was chosen because it showed a high cell death percentage (~50%), which should enhance the relative influence of mechanical stress on CisPt-carnosine complex during experimentation.

### 2.4. PBMCs

Whole blood was obtained from a healthy donor after obtaining informed consent and institutional ethics approval. For every experiment, 10 mL of blood was collected from a healthy donor, for a total of 100 mL (Genuine Research Center, Egypt). PBMCs isolation was done using Histopaque (Sigma-Aldrich, USA). PBMCs were finally resuspended in a complete medium consisting of RPMI 1640 containing 10% heat-inactivated FBS (Lonza, USA) and 5% penicillin-streptomycin (Lonza, USA). HepG2 cells were seeded overnight at the aforementioned cell count. For preliminary testing, freshly isolated PBMCs were added for a range of ratios of PBMCs: HepG2 cells, namely, 5.8 : 1, 2.9 : 1, 1.4 : 1, and 0.7 : 1, to validate present methodology against prior literature [15]. For the current study, a final ratio of 3 : 1 was chosen as it is a common ratio used in the scientific literature [15, 22].

### 2.5. Mechanical Stress

Mechanical stress was applied on HepG2 cells only, PBMCs only, and PBMCs/HepG2 cocultured cells using an Orbi-Shaker CO_2_ (Benchmark Scientific, USA). The shaker produces a horizontal and circular motion, with an orbit diameter of 19 mm for aeration and mixing over a speed range of 30 to 300 rpm at 1 rpm increments. For preliminary testing, a range of 100 to 300 rpm was assessed. Shaking was done for 24 hours. For the current study, a final speed of 300 rpm was chosen; this was based on the early trials that showed no effect below 300 rpm. Kraiss et al. and Dardik et al. had previously used similar orbital shakers to exert shear stress on cultured cells and estimated the maximal shear stress to be 11.5 dyn/cm^2^ which is equivalent to 1.15 Pascals (Pa) [23, 24]. In this study, with our used parameters (300 rpm and orbital rotation radius of 0.95 cm), the maximal shear stress was calculated with the same equation and estimated to be 14.5 dyn/cm^2^ (1.45 Pa) [23, 24]. The used values were the same theoretical values reported by Kraiss et al. [23], wherein the viscosity of the medium *η* was estimated to be 0.0075 poise and the density of the medium *ρ* 1.0 g/mL.

### 2.6. Carnosine

Carnosine (*β*-alanyl-L-histidine dipeptide) powder (Sigma-Aldrich, UK) was dissolved in sterile water to prepare a 1 mg/mL stock solution which was then stored at 4°C in an amber bottle. Different carnosine : CisPt molar ratios (1 : 1, 1.5 : 1, 2 : 1, 2.5 : 1, and 3 : 1) were prepared from the stock solution with fixed CisPt concentration of 8 *μ*g/mL, and these solutions were incubated for 24 hours at 4°C. 24 hours before exposure to the different solutions and/or mechanical stress, HepG2 cells were seeded at 1 × 10^4^ cells/well under the abovementioned conditions. Old media were discarded and the different solutions were added to the wells before incubation for additional 24 hours at 37°C and 5% CO_2_ prior to conducting the MTT assay (cell viability assay). The ratio that resulted in the highest cytotoxic effect, with simultaneous exposure to mechanical stress (carnosine : CisPt, 2.5 : 1), was then compared with CisPt alone for its cytotoxic activity with and without applying mechanical stress.

### 2.7. Cell Viability Assay

MTT assay was used to determine the number of viable cells after exposure to the different factors. MTT is a yellow chemical, 3-(4, 5-dimethylthiazolyl-2)-2, 5-diphenyltetrazolium bromide, classified as a tetrazolium compound which reacts with mitochondrial dehydrogenase enzymes in viable cells to produce a purple formazan precipitate [21]. After exposure to the various factors, cells seeded in 96-well plates had their old media discarded and were replenished with 100 *μ*L complete media. Exactly 20 *μ*L MTT (5 mg/mL) (Serva, Germany) were added to each well and incubated for 3 hours. Then media were discarded from all wells and 100 *μ*L DMSO (Sigma-Aldrich, USA) were added to solubilize the purple precipitate when present. Absorbance (*A*) was measured using a FLUOstar OPTIMA microplate reader (BMG LabTech, Germany) at 595 nm [21]. Cell viability was calculated as cell proliferation % = [*A* (sample) −* A* (control)]/*A* (control) × 100. For all experiments, control groups were either HepG2 cells cultured in complete media, complete media with the solvent used to dissolve CisPt, or complete media with carnosine solution or with the solvent used to dissolve carnosine.

### 2.8. RT-PCR

RNA was extracted by Trizol (Invitrogen, USA) from HepG2 stressed and control cells. RNA (0.5 *μ*g/*μ*L) was used to synthesize cDNA using random primers following a RevertAID First Strand cDNA synthesis kit (Fermentas, USA). Then, the relative expression of BAX, XIAP, HSP70, and Caspase-3 genes was examined using a semiquantitative polymerase chain reaction (RT-PCR). Expression levels of the 4 genes were compared to *β*-actin. Forward and reverse PCR primer sequences were BAX forward primer, 5′-ATGGACGGGTCCGGGGAG-3′, and reverse primer, 5′-TCAGAAAACATGTCAGCTGCC-3′ [25]; XIAP forward primer, 5′-TTTTCCAAGTGGTAGTCCTGTTTC-3′, and reverse primer, 5′-GCTGAGTCTCCATATTGCCATCTA-3′ [26]; HSP70 forward primer, 5′-AGGCGGACAAGAAGAAGGTGCT-3′, and reverse primer, 5′-TGGTACAGTCCGCTGATGATGG-3′ [27]; Caspase-3 forward primer, 5′-AGAGGGGATCGTTGTAGAAG-3′ [28], and reverse primer, 5′-GTTGCCACCTTTCGGTTAAC-3′; and *β*-Actin forward primer, 5′-CGTGGGCCGCCCTAGGCACCA-3′, and reverse primer, 5′-TTGGCCTTAGGGTTCAGGGGGG-3′ [22]. Expected cDNA amplicon sizes are BAX 305 bp, XIAP 132 bp, HSP70 145 bp, Caspase-3 305 bp, and *β*-Actin 243 bp. Earlier cell viability tests were destructive to the cells; thus the cell culture experiments were repeated for the RT-PCR and immunoblotting assays. Consequently, only 2 or 3 samples were used for each of the 9 groups assessed, namely, negative control, UV-treated (positive control), CisPt alone, PBMCs alone, CisPt + PBMCs, mechanical stress alone, mechanical stress + CisPt, mechanical stress + PBMCs, and mechanical stress + CisPt + PBMCs. These tests were only a supplemental indicator of cell death.

### 2.9. Immunoblotting

Proteins were extracted from test and control cells using Laemmli buffer containing a cocktail of protease inhibitors (Fermentas, cat. number R1329) and were quantified using a BCA protein assay kit (Pierce, USA, cat. number 23225). Protein extracts were separated on 12% SDS-PAGE gels and transferred onto a PVDF membrane. The membrane was blocked with 5% nonfat dry milk and incubated with a PARP-1 antibody (Cell Signaling Technology, USA) diluted in 3% nonfat dry milk overnight at 4°C [29]. The chosen antibody detects full-length and cleaved PARP-1 proteins (113 and 89 KDa, resp.) characteristic of apoptosis at those specific cleavage sites. The secondary antibody (i.e., alkaline phosphatase anti-Rabbit antibody) (KPL, USA) was diluted in 3% nonfat dry milk and incubated at room temperature for 30 minutes. PhosphaGlo substrate was used to detect chemiluminescent signals (KPL, USA). The membranes were stripped using a buffer containing *β*-mercaptoethanol and incubated for 15 min at 65°C. After stripping the membrane, the *α*-tubulin (loading control) (Pierce, USA) primary antibody was added (55 KDa) and the immunoblot was repeated.

### 2.10. UV Treatment

HepG2 cells were exposed for 10 minutes to ultraviolet light with a wavelength of 253.7 nm inside the biosafety cabinet (Airstream Class II Biological Safety Cabinet, E-Series, Esco) after which cells were lysed for RT-PCR or Western blot analyses [30]. These cells served as a positive control for apoptotic cell death-indication.

### 2.11. Statistical Analysis

**Figure 1:**
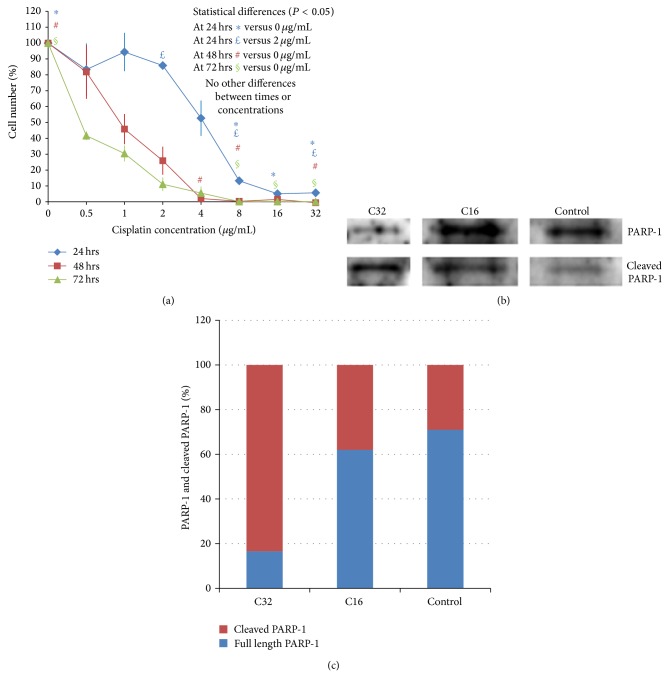
The effect of CisPt (0.5–32 *μ*g/mL) on HepG2 cells. (a) Cell viability of HepG2 cells after exposure to CisPt (0.5–32 *μ*g/mL) for 24, 48, and 72 hours as per MTT assay. The data are presented as a mean of at least three independent experiments. Error bars represent one standard error of the mean (*P* < 0.05). (b) Western blot analysis showing full-length and cleaved PARP-1 expression in HepG2 cells after exposure to different factors. UV = HepG2 cells exposed to ultraviolet light, C32 = cells exposed to CisPt 32 *μ*g/mL, C16 = cells exposed to CisPt 16 *μ*g/mL, and Control = HepG2 cells not exposed to any factor (negative control). (c) The percentage of full-length PARP-1 and cleaved PARP-1 as compared to the total PARP-1. The extent of PARP-1 cleavage was quantified by measuring relative intensities of the cleaved PARP-1 bands in the Western blot of Figure 1(b) and comparing them to intensity of the full-length PARP-1.

First, for cell viability % versus cisplatin concentrations (Figure 1(a)), a one-way ANOVA was used with Tamhane's T2* post hoc* analysis (*P* < 0.05) to identify specific pairwise differences. Tamhane's method was chosen since the Levene test showed nonhomogeneity of variances between groups (*P* < 0.001).

**Figure 2:**
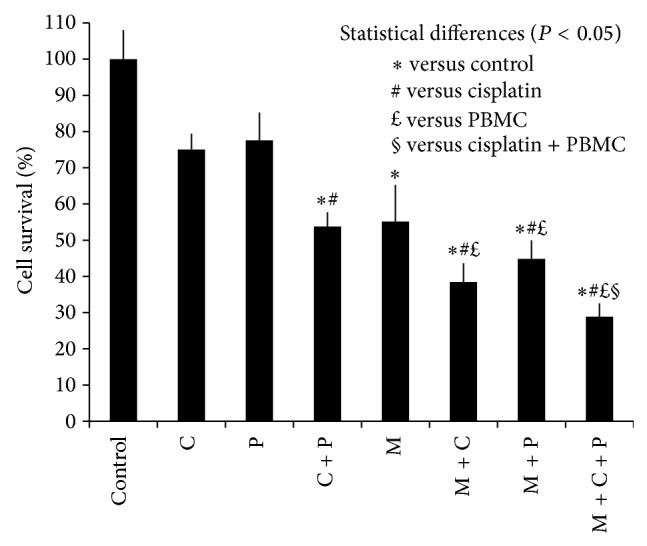
HepG2 cell survival rate after exposure to different factors for 24 hours exposure. Control = cells not exposed to any factor (negative control), C = CisPt 2 *μ*g/mL, P = PBMCs, and M = mechanical stress. Error bars represent one standard error of the mean (*P* < 0.05).

Second, for cell viability versus chemical and/or mechanical stress factors (Figure 2), preliminary analysis using one-way ANOVA with Tamhane's T2* post hoc* analysis (*P* < 0.05) showed no effect on cell death due to which one of the 3-day experiments was performed; that is, 19 of 24 pairwise comparisons of the same parameter between the 3 days showed no difference (0.077 ≤ *P* ≤ 1.000). Thus, it was then appropriate to group into a one-way ANOVA all data across the 3 test days from each well to perform the final pairwise comparisons with Tamhane's* post hoc* test (*P* < 0.05), which are presented here. Tamhane's method was used for the preliminary and final analyses, since the Levene test showed nonhomogeneity of variances between groups (*P* < 0.001).

**Figure 3:**
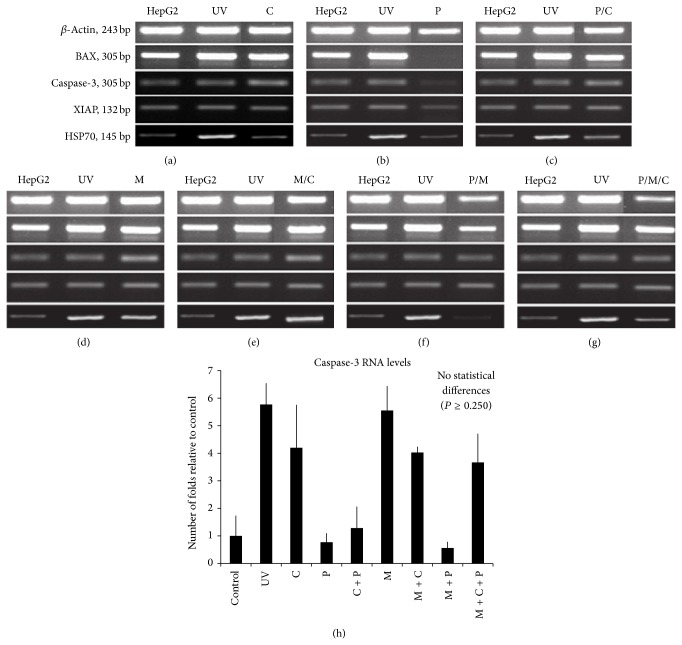
RNA expression of apoptosis-related genes BAX, Caspase-3, XIAP, HSP70, and the internal control *β*-Actin after exposure of HepG2 cells to different factor(s). Columns from left to right are unstressed cells (negative control), HepG2 cells exposed to UV radiation (positive control), and HepG2 cells exposed to different factor(s). (a) HepG2 cells exposed only to CisPt 2 *μ*g/mL for 24 hours. (b) HepG2 cells cocultured with PBMCs in 3 : 1 ratio (PBMCs : HepG2 cells) for 24 hours. (c) HepG2 cells cocultured with PBMCs in 3 : 1 ratio (PBMCs : HepG2 cells) and simultaneously exposed to CisPt 2 *μ*g/mL for 24 hours. (d) HepG2 cells exposed only to mechanical stress at 300 rpm for 24 hours. (e) HepG2 cells simultaneously exposed to CisPt 2 *μ*g/mL and mechanical stress at 300 rpm for 24 hours. (f) HepG2 cells cocultured with PBMCs in 3 : 1 ratio (PBMCs : HepG2) and simultaneously exposed to mechanical stress at 300 rpm for 24 hours. (g) HepG2 cells cocultured with PBMCs in 3 : 1 ratio (PBMCs : HepG2) and simultaneously exposed to CisPt 2 *μ*g/mL plus mechanical stress at 300 rpm for 24 hours. (h) Caspase-3 RNA levels as indicated by the number of folds relative to the Control. Control = cells not exposed to any factor (negative control), C = CisPt, P = PBMCs, M = mechanical stress, and UV = cells exposed to ultraviolet (positive control). Error bars represent one standard error of the mean.

Third, for Caspase-3 RNA levels versus chemical and/or mechanical stress factors (Figure 3(h)), a one-way ANOVA was used with Tamhane's T2* post hoc* analysis (*P* < 0.05) to identify specific pairwise differences. Although the Levene test showed homogeneity of variances (*P* = 0.175) permitting the use of a less stringent* post hoc* test, Tamhane's method was still chosen, since it is a more conservative approach and the numbers of samples per test group were not equal, thus not allowing for the use of a less conservative* post hoc* test like Tukey's HSD.

Fourth, for cell viability % versus carnosine : cisplatin ratio (Figure 5), the raw data were initially corrected by comparison to blank readings. The corrected data were then analyzed using a one-way ANOVA with Tukey's HSD test (*P* < 0.001) for pair-wise comparisons. Tukey's method was used, because the Levene test showed homogeneity of variances between groups (*P* = 0.227).

Finally, for cell viability % versus exposure to carnosine and cisplatin with or without mechanical stress (Figure 6), the raw data were initially corrected by comparison to blank readings. The corrected data were then analyzed using one-way ANOVA with Tukey's HSD test (*P* < 0.001) for pairwise comparisons. Tukey's method was appropriate to use, since each test group had exactly the same number of samples (*n* = 8).

## 3. Results

### 3.1. The Effect of Different Parameters (or Stress Factors) on Killing HepG2 Cells

The percent of survival of cancer cells for the 8 test groups that addressed the effect of mechanical stress on the efficacy of CisPt as per MTT assay is shown in Figures 1(a) and 2. Although exposing cells to mechanical stress has accelerated death of cells, more cancer cells were killed using mechanical stress with or without CisPt and PBMCs, when compared to CisPt, PBMCs, or CisPt + PBMCs. Specifically, for the 8 test groups, there were a total of 28 possible pairwise statistical comparisons, which yielded 13 statistical differences (*P* < 0.05). Almost all of these statistical differences involved mechanical stress (11 of 13 cases). For all the RT-PCR results (Figures 3(a)–3(h)), the assay is regarded as semiquantitative and all the bands' intensities were first normalized by comparing them with the relevant internal control band, prior to comparison with bands for other conditions.

#### 3.1.1. Cytotoxicity of CisPt as a Single Stress Factor

CisPt killed HepG2 cells in a dose-dependent manner with the lowest survival at the highest concentrations (8–32 *μ*g/mL) and longest incubation times (48–72 hours) (Figure 1(a)). CisPt at 32 and 16 *μ*g/mL resulted in a higher ratio of cleaved PARP-1 to full-length PARP-1 than the control HepG2 cells (Figures 1(b) and 1(c)).

Cell survival rate under CisPt alone was 75%, which was not statistically different from the control, PBMCs alone, or mechanical stress alone, but it was statistically higher than cell survival when using CisPt + PBMCs, mechanical stress + CisPt, mechanical stress + PBMCs, or mechanical stress + CisPt + PBMCs (Figure 2).

At the RNA level, BAX, Caspase-3, and XIAP expression increased to levels similar to UV results, while HSP70 maintained levels similar to preexposure to CisPt (Figure 3(a)).

#### 3.1.2. The Effect of PBMCs as a Single Stress Factor

Cell survival rate for PBMCs alone was 78%, which was not statistically different from the control, CisPt alone, CisPt + PBMCs, or mechanical stress alone, but it was statistically higher than cell survival when using mechanical stress + CisPt, mechanical stress + PBMCs, or mechanical stress + CisPt + PBMCs (Figure 2).

At the RNA level, BAX, Caspase-3, XIAP, and HSP70 expression showed a decline of all 4 genes (Figure 3(b)). Concerning Caspase-3, although there was no statistical difference, likely due to small sample size (Figure 3(h)), there was an observed difference in the Caspase-3 level, suggesting a decline (Figure 3(b)).

#### 3.1.3. The Effect of CisPt and PBMCs

Cell survival rate for CisPt + PBMCs was 54%, which was statistically lower than the control and CisPt alone and statistically higher than mechanical stress + CisPt + PBMCs (Figure 2).

At the RNA level, BAX and Caspase-3 expression levels were slightly higher (Figure 3(c)), while XIAP and HSP70 expression levels were comparable before and after stressing HepG2 cells with both CisPt and PBMCs.

#### 3.1.4. The Effect of Mechanical Stress as a Single Stress Factor

Cell survival for mechanical stress alone was 55%, which was statistically lower than control, but was not statistically different compared to any other test group (Figure 2).

RNA steady state levels of BAX and Caspase-3 were comparable to those determined for UV-treated HepG2 cells when compared to untreated cells, but XIAP was slightly higher whereas HSP70 expression levels were not affected by the treatment (Figure 3(d)).

#### 3.1.5. The Effect of Mechanical Stress and CisPt

Cell survival rate for mechanical stress + CisPt was 38%, which was statistically lower than control, CisPt alone, and PBMCs alone, but was not statistically different from other test groups (Figure 2).

At the RNA level, BAX and Caspase-3 were upregulated, and a slight increase occurred in the levels of XIAP gene expression, but these were comparable to cells exposed to UV light, while RNA expression of HSP70 was similar to the control (Figure 3(e)).

#### 3.1.6. The Effect of Mechanical Stress and PBMCs

Cell survival rate for mechanical stress + PBMCs was 45%, which was statistically lower than control, CisPt alone, or PBMCs alone, but was not statistically different from other test groups (Figure 2).

At the RNA level, BAX, XIAP, and HSP70 expressions increased while that of Caspase-3 decreased when compared to those of the untreated cells and cells that received UV light treatment (Figure 3(f)).

#### 3.1.7. The Effect of Mechanical Stress, CisPt, and PBMCs

Cell survival rate for mechanical stress + CisPt + PBMCs was only 29%, which was statistically lower than control, CisPt alone, PBMCs alone, or CisPt + PBMCs, but it was not statistically different from any of the other test groups involving mechanical stress (Figure 2).

At the RNA level, BAX and Caspase-3 expressions were upregulated, while XIAP and HSP70 RNA were comparable to the control cells whether treated with UV light or untreated (Figure 3(g)).

#### 3.1.8. Cell Death: Morphology and Mechanism

**Figure 4:**
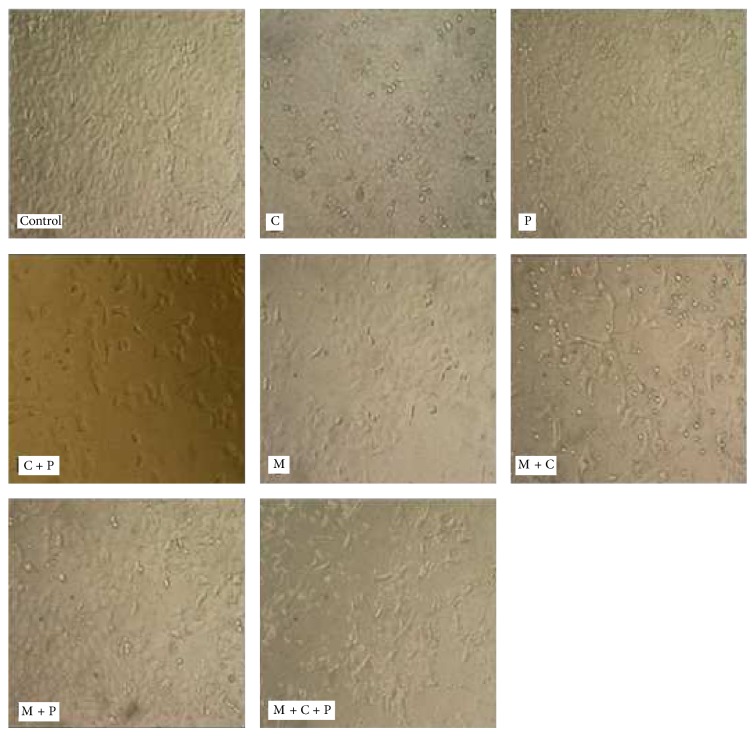
Morphological changes at 200x magnification for HepG2 cells exposed to different factors. Control = cells not exposed to any factor (negative control), C = CisPt, P = PBMCs, and M = mechanical stress.

Irrespective of the type of treatment, dying HepG2 cells always exhibited a rounded shape and lost attachment to the cell culture plate when compared to the control cells with an epithelial shape (Figure 4). However, the highest rate of HepG2 cell death was caused, as predicted by the mathematical model proposed by Klika and Maršík [10], when mechanical stress, CisPt, and PBMCs were combined (Figure 2).

Concerning PARP-1 cleavage, an indication of apoptosis, CisPt 32 and 16 *μ*g/mL resulted in a higher ratio of cleaved PARP-1 to full-length PARP-1 than the control HepG2 cells (Figures 1(b) and 1(c)).

Our results suggest that programmed cell death that was induced in HepG2 cells when exposed to UV, CisPt, mechanical stress, mechanical stress + CisPt, and mechanical stress + CisPt + PBMCs appears to involve Caspase-3. On the other hand, exposure of HepG2 cells to the conditions PBMCs, PBMCs + CisPt, and PBMCs + mechanical stress did not cause Caspase-3 RNA levels elevation (Figures 3(a)–3(h)). Hence, HepG2 cell death after exposure to the aforementioned conditions does not seem to be associated with Caspase-3.

### 3.2. The Possible Mechanism by Which Mechanical Stress Increases the Efficacy of CisPt

In an attempt to understand the nature of action imposed by mechanical stress resulting in the augmentation of CisPt efficiency, we hypothesized that mechanical stress frees CisPt sequestered in the cytoplasm and mediates its translocation into the nucleus, its site of action. To test our hypothesis, we incubated CisPt with carnosine for 24 hours to allow, if feasible, complex formation in culture. We have recently reported that carnosine binds to OxPt, which is a platinum-based drug similar to CisPt [18]. The survival rate of HepG2 cells treated with these complexes in the presence and absence of mechanical stress was also monitored via MTT assay (see below).

#### 3.2.1. The Effect of CisPt-Carnosine Complex on HepG2 Cell Survival

**Figure 5:**
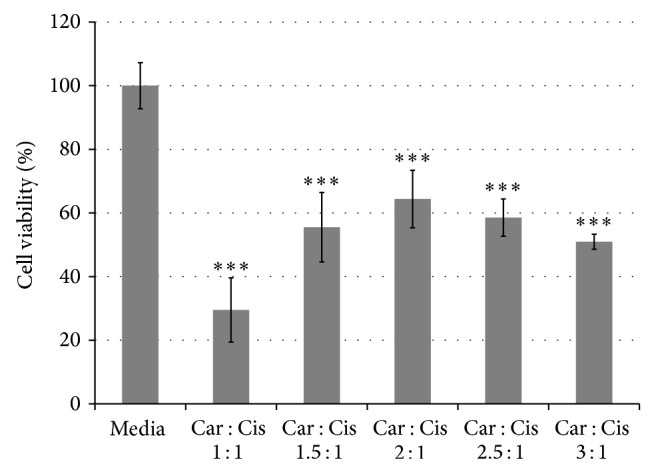
Different carnosine : CisPt molar ratios cytotoxic effect on HepG2 cells for 24 hours as per MTT assay. The data are presented as a mean of at least three independent experiments (mean ± SD). Left to right: Media = control HepG2 cells. Car : Cis 1 : 1 = HepG2 cells exposed to CisPt 8 *μ*g/mL and carnosine in 1 : 1 molar ratio. Car : Cis 1.5 : 1 = HepG2 cells exposed to CisPt 8 *μ*g/mL and carnosine in 1.5 : 1 molar ratio. Car : Cis 2 : 1 = HepG2 cells exposed to CisPt 8 *μ*g/mL and carnosine in 2 : 1 molar ratio. Car : Cis 2.5 : 1 = HepG2 cells exposed to CisPt 8 *μ*g/mL and carnosine in 2.5 : 1 molar ratio. Car : Cis 3 : 1 = HepG2 cells exposed to CisPt 8 *μ*g/mL and carnosine in 3 : 1 molar ratio (error bars represent the standard deviation). The *P* values shown are the values of the mean of every condition as compared to the control cells (^***^ *P* < 0.001).

HepG2 cells were cultured in a mixture of carnosine and CisPt. As previously mentioned, the mixture was left for 24 hours in order to allow a fixed amount of CisPt (8 *μ*g/mL), together with different molar concentrations of carnosine (1 : 1, 1.5 : 1, 2 : 1, and 2.5 : 1), to complex before adding the mixture to the HepG2 cells. Upon culture in these different mixtures, the cell viability varied between ~30% and 65%, where it increased from 1 : 1 to 2 : 1, and then slightly decreased afterwards in 2.5 : 1 and 3 : 1 molar ratios of carnosine : CisPt (Figure 5).

#### 3.2.2. The Effect of CisPt-Carnosine and Mechanical Stress on HepG2 Cell Survival

**Figure 6:**
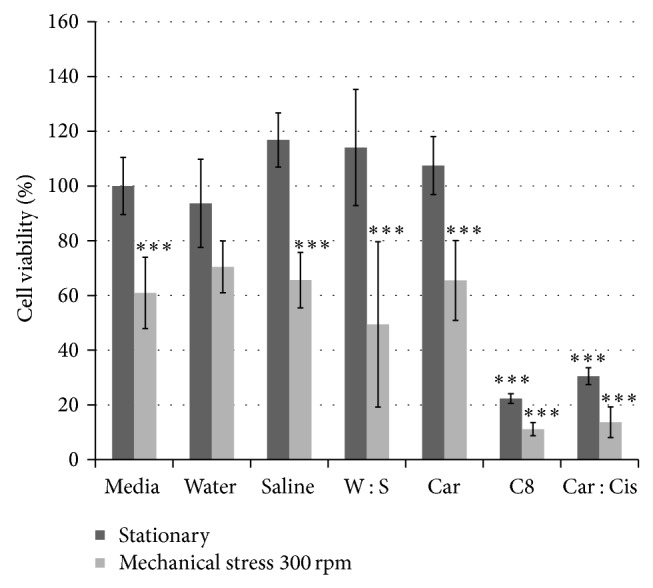
Cell viability of HepG2 cells after exposure to CisPt (8 *μ*g/mL) and carnosine (1 : 3 molar ratio) with and without mechanical shaking (300 rpm) for 24 hours as per MTT assay. The data are presented as a mean of at least three independent experiments (mean ± SD). Left to right: Media = control HepG2 cells. C8 = HepG2 cells exposed to CisPt 8 *μ*g/mL. C : C = HepG2 cells exposed to CisPt 8 *μ*g/mL and carnosine in 1 : 3 molar ratio. Car = HepG2 cells exposed to carnosine. Water = HepG2 cells exposed to water (carnosine control). Saline = HepG2 cells exposed to saline (CisPt control). W : S = HepG2 cells exposed to water and saline (C : C control). (Error bars represent the standard deviation.) The *P* values on the bars denote the significant differences as compared to the control cells that were seeded in a stationary plate and with media not supplemented with any chemical agent (^***^ *P* < 0.001).

In the stationary plate, the drug alone showed about a 10% higher cytotoxic effect than in combination with carnosine (Figure 6). On the other hand, the level of cytotoxicity of CisPt on HepG2 cells increased in the presence of carnosine and mechanical stress as indicated by the decrease in cell viability (Figure 6). These results suggest that mechanical stress appears to act as a death catalyst.

## 4. Discussion

### 4.1. General Findings

**Figure 7:**
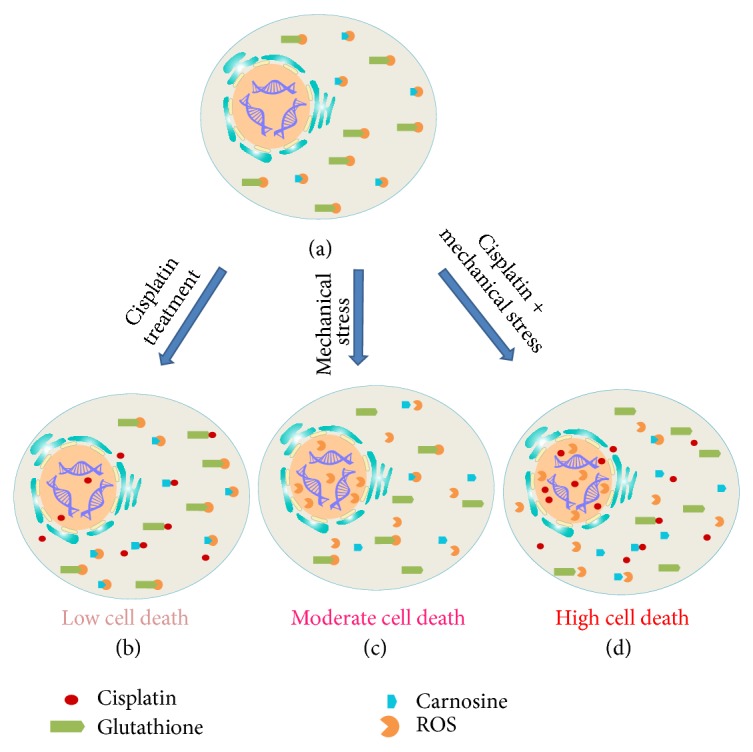
Schematic diagram for the effect of CisPt, mechanical stress, and CisPt combined with mechanical stress on HepG2 cells. (a) HepG2 cells without exposure to any stress factors have reactive oxygen species (ROS) bound to glutathione or to carnosine. (b) Upon addition of CisPt, low cell death results probably due to the complexation of some CisPt molecules to carnosine or glutathione in the cytoplasm. (c) Upon exposure of HepG2 cells to mechanical stress, complexes of ROS-glutathione and ROS-carnosine are disassembled; hence ROS is free to enter the nucleus and cause moderate cell death. (d) In the presence of CisPt and mechanical stress, many CisPt-Car, CisPt-glutathione, ROS-carnosine, and ROS-glutathione complexes are disassembled and many CisPt and ROS molecules enter the nucleus resulting in high cell death.

This investigation shows that combining mechanical stress with both CisPt and PBMCs effectively enhances the death of HepG2 cancer cells in culture (Figure 2). Moreover, the application of mechanical stress alone or in combination with either CisPt or PBMCs was also effective in destroying cancer cells, though to a slightly lesser extent (Figure 2). Conversely, CisPt and PBMCs alone or combined were not particularly noteworthy in this regard (Figure 2). These data agreed with the trends observed for RNA steady state levels of Caspase-3 (Figures 3(a)–3(g)). Further, when HepG2 cells were treated with CisPt-carnosine complexes, cell death was slightly compromised compared to treatment with CisPt alone (Figure 5). However, the effect of CisPt on cell death was restored upon introducing mechanical stress and was comparable to the effect of CisPt (uncomplexed with carnosine) + mechanical stress (Figure 6). Taken together, the data suggest that mechanical stress boosted the efficacy of CisPt. In an attempt to understand the underlying mechanism of action of mechanical stress and CisPt on cell death, we suggest the model depicted in Figure 7. As previously reported, when HepG2 cells are exposed to CisPt alone, most of the CisPt is retained in the cytoplasm and binds to endogenous ligands such as glutathione and carnosine [16, 17]. Hence, most of the CisPt is not at its pharmacological target site of action and will have a limited cytotoxic effect (Figure 7(b)). Upon the addition of mechanical stress together with CisPt, cytoplasmic CisPt molecules will be mobilized into the nucleus, which causes a higher cytotoxic effect (Figure 7(d)). This model attempts to explain the results as per MTT assay (Figures 2, 5, and 6), and more cytoplasmic ligands should be further investigated in order to better understand the reasons behind entrapping CisPt in the cell cytoplasm. Such knowledge would pave the way for developing new treatments to combat CisPt resistance. To the authors' knowledge, this is the first* in vitro* report of cancer cell death caused by various combinations of mechanical stress, CisPt, and/or PBMCs. Consequently, mechanical stress's potential as a clinical cancer treatment warrants further investigation.

### 4.2. Comparison to Prior Studies

It is well known that dynamic stress or mechanical stimulus plays a major role in cell regulation [31–33]. However, the coupling effect resulting from the interaction between the mechanical stress and the cells in the presence of, for instance, drugs, hormones, or vitamins is still not understood. A prior experimental study on lung cancer examined mechanical “stretch” (i.e., tensile stress) combined with anticancer drugs such as CisPt, Doxorubicin, Paclitaxel, Zactima, and an experimental drug [34]. The mechanical stress approximated human lung expansion and contraction at 20% maximum strain and 15 cycles/minute [34]. They reported that mechanical “stretch” alone triggered lung cancer cell death, whereas when combined with CisPt the efficacy of the drug decreased [34]. This result was unlike present data, which showed that mechanical stress + CisPt is more effective in killing cancer cells than mechanical stress alone, as seen in the control and several other test groups. This divergent result compared to the current report may be due to the different type of “shear” stresses and cancer cells used. However, the previous authors did show that mechanical stress + Zactima increased cancer cell death [34], which agrees with the present general finding that mechanical stress can enhance anticancer drug effectiveness.

Another experimental investigation examined the effect of mechanical stress on the shape of nonmetastatic murine mammary carcinoma tumors [35]. The investigators coembedded individual cancer cells with fluorescent microbeads and used confocal microscopy to obtain the 3D distribution of the microbeads that surrounded growing tumor spheroids. They then applied compressive pressures ranging from 0 to 60 mmHg for 17 hours. Finally, they converted changes in microbead density to strain as an estimate of compressive mechanical stress around the tumor spheroids. They found a strong positive linear correlation (*r* ^2^ = 0.75) between mechanical stress and tumor cell death, as indicated by the shape of the tumors. They attributed this effect to the suppression of cancer cell proliferation and induction of cell apoptosis in areas of high mechanical stress [35]. This agrees with the current study's finding that mechanical stress alone can statistically reduce cancer cell survival to 55% compared to the control group. The mechanism through which mechanical stress contributes to cancerous cell death remains to be determined. However, the cumulative results of the current study suggest that mechanical stress acts as a dissociating agent that causes endogenous antioxidant molecules, such as carnosine, to release their binding partners. Endogenous antioxidants are known to interact with reactive oxygen species (ROS), which are implicated in the development of cancer [36], as well as to bind to CisPt [16] and OxPt [18]. We propose that in the absence of the Pt-based drug, CisPt, mechanical stress is most likely releasing the cytosol ROS and making it available to act on the nucleus (Figure 7(c)).

A previous theoretical study using a linear nonequilibrium thermodynamic mathematical model suggested that dynamic mechanical stress plus anticancer drugs in the presence of healthy cells leads to high cancer cell death [10]. The reason may be that some biochemical processes, such as dissociating the complexation of an anticancer drug with cytoplasmic ligands (i.e., carnosine), need to be triggered by mechanical stimulation and, conversely, some mechanical processes may need the energy input offered by biochemical processes. In other words, there is a coupling effect between biochemical and mechanical processes [10]. The previous study also showed that dynamic, rather than static, mechanical stress is required to influence biochemical processes [10]. These findings partially explain and concur with the present data. Specifically, it is possible that the current biochemical activity of the anticancer drug CisPt can be enhanced by the dynamic mechanical stimulation of horizontal circular motion. Conversely, the theoretical modeling of the prior investigation is confirmed by the current study, as it is the first to experimentally quantify and validate the effect on cancer cells of combining dynamic mechanical stress with CisPt in the presence of healthy immune cells (PBMCs).

### 4.3. Clinical Implications

Two main limitations that are associated with CisPt use for cancer patients are the resultant side effects and resistance [2–6]. Side effects arise due to an inherent chemical property of CisPt; CisPt is not specific to cancer cells only [2–6]. In this study, we aimed to address CisPt limitations by introducing factors that would enhance the effect of CisPt in killing more cancer cells* in vitro*. However, this study was not concerned with improving CisPt cell specificity. The approach used in this study was to apply mechanical stress and PBMCs, healthy immune cells, that would be synergistic with CisPt and result in higher cancer cell death; hence the applied dose would eventually be lower, theoretically followed by fewer side effects experienced by cancer patients. Mechanical stress was also introduced to overcome resistance of the cells to CisPt and aid in translocating CisPt to the nucleus, a mechanism that culminates in higher cancer cell death percentages.

The pharmacological target of Pt-based anticancer drugs such as CisPt, carboplatin, and OxPt is the nuclear DNA [37]. Concerning the effect of mechanical stress on cancer cells, current results show that the mere presence of mechanical stress increased cancer cell death compared to the control (Figures 2 and 6). When combined with CisPt alone, PBMCs alone, or CisPt + PBMCs, mechanical stress further statistically reduced cancer cell survival. However, the use of CisPt or PBMCs alone did not statistically reduce cancer cell survival below control levels. Only when CisPt was combined with PBMCs were cancer cells destroyed below control levels, but this was not different from the activity of mechanical stress alone. Consequently, the individual effectiveness of CisPt and PBMCs as a cancer treatment protocol is drawn into question by these results, whereas the potential effectiveness of mechanical stress as a primary or supplementary treatment strategy has been demonstrated. Similarly, while some studies have demonstrated an association between mechanical stress and cancer cell proliferation and invasiveness [38, 39], other studies have shown a positive linear relationship between cell death and mechanical stress [35].

CisPt, like all Pt-based drugs, kills cancer cells through the formation of stable Pt-DNA adducts at the nitrogen in position 7 of guanine (N7). However, CisPt has been shown to have a higher affinity for amino acids and cytoplasmic ligands, which are sulfur donors, than to DNA, which is a nitrogen donor, that is located in the nucleus [40]. Kasherman and colleagues [16] have used whole cell extract as a model for the cytoplasm to demonstrate the binding between CisPt and the cytoplasmic ligand glutathione (GSH). Their study concluded that GSH, a tripeptide, composed of L-cysteine, L-glutamic acid, and glycine, which functions as an endogenous antioxidant, is not responsible for sequestering most of the cellular CisPt in the cytoplasm. Their data suggested the presence of other intracellular ligands contributing to the decreased efficacy of CisPt due to its entrapment in the cytosolic compartment of the cell [16]. It is because of these reasons that we decided to examine the interaction of CisPt with carnosine, which is another endogenous antioxidant that has been shown to interact with OxPt [18], in order to determine the role mechanical stress might play in increasing the drug efficacy.

In an attempt to understand the underlying mechanism of action of CisPt and mechanical stress, Figure 7(b) demonstrates that when HepG2 cells are exposed to CisPt alone, most of the CisPt is retained in the cytoplasm and binds to endogenous ligands such as glutathione and carnosine. Hence, most of the CisPt is not at its target site of action and will have a limited cytotoxic effect. Upon the addition of mechanical stress together with CisPt (Figure 7(d)), cytoplasmic CisPt molecules will be mobilized into the nucleus, which causes a higher cytotoxic effect. The suggested model (Figure 7) attempts to explain the results obtained from the conducted cytotoxicity assays (Figures 2, 5, and 6). Nevertheless, more ligands should be further investigated in order to better understand the mechanism of CisPt resistance. Such research in turn would pave the way for developing new treatments that would combat CisPt resistance.

In the current study, we present data supporting the hypothesis that the complexation of CisPt with the cytoplasmic ligand carnosine was compromised, most likely, in response to mechanical stress application. The CisPt-carnosine complexes, as depicted in Figure 6, were less cytotoxic than CisPt alone, similar to the effect observed upon treating cells with OxPt-carnosine complexes [18]. Based on these results, we propose mechanical stress as a promising supplementary treatment modality for cancer patients who develop resistance to chemotherapy. However, the optimal method for applying mechanical stress* in vivo* should be studied thoroughly. Drug resistance to Pt-based drugs, whether intrinsic or acquired, is responsible for the failure of chemotherapy in treating many cancer patients [41, 42]. Intrinsic resistance to chemotherapy indicates tumors that do not respond to the drug from the beginning of treatment, whereas acquired resistance denotes tumors that develop resistance to a particular anticancer drug despite sensitivity to it during the early stages of treatment [43]. Although the mechanism through which tumor cells acquire CisPt resistance is not yet well understood, it may share some commonality with resistance resulting from exposure to OxPt. Recently, the current authors have demonstrated unequivocally that complexation of OxPt with carnosine is partly responsible for the accumulation of OxPt in the cytoplasm away from the nucleus, its pharmacological target site [18]. Further experiments are required to confirm this mechanism and to characterize these complexes, as well as their formation and deformation mechanisms.

Regarding cancer cell death pathways, current HepG2 cells appeared to employ several pathways in response to the applied factors (Figures 1(b), 1(c), and 3). PARP-1 apoptotic cleavage was involved in the death of HepG2 cells when only CisPt or PBMCs was used (Figures 1(b) and 1(c)). Interestingly, when mechanical stress was combined with CisPt and/or PBMCs, cell death appeared to exclude the involvement of PARP-1 apoptotic cleavage (Figures 1(b) and 1(c)). In this case, BAX, which is highly upregulated at the RNA level, may be implicated in HepG2 cell death (Figures 3(a)–3(g)). Both BAX and PARP-1 were involved in apoptotic cell death. The proapoptotic protein BAX was essential for the activation of apoptosis, whereas PARP-1 was likely the perpetrator of apoptotic cell death [44]. In fact, PARP-1 cleavage, which was mediated by active Caspase-3, occurred in limited cases of apoptosis. Given that apoptosis is a multipathway program, further experiments are required to confirm the involvement of BAX protein, rather than RNA levels, in a death pathway that utilizes factors other than PARP-1 as a killing effect. Additional experiments should also test other death mechanisms, such as necrosis and atrophy, which may also be responsible [44].

The current study's main aim was to provide a “proof of principle” regarding the effect of mechanical stress on the survival of cancerous cells, rather than to prematurely propose a clinically useful device or treatment using mechanical stress. Such an application will require time for development by future researchers. However, the authors do envision the possibility of using whole body vibration or whole limb vibration in the treatment of cancer, but only if tolerable by the patient and if there has already been significant metastatic spread of cancer throughout the body or specific limb. In the case of a single localized tumor or several localized tumors, targeted vibration could potentially be accomplished using a probe with a small vibrating head or tip that is either applied externally to the tumor or inserted directly into the tumor. This would also eliminate or minimize negative mechanical stress effects on normal cells. Several commercially available devices that use mechanical vibration for physical rehabilitation, massage therapy, or dentistry may serve as rudimentary conceptual prototypes which could be further developed for clinical use in tumor treatment. Previously mechanical strain was applied to cells in culture, namely, osteoblasts, for bone-related conditions [45], and, concerning tumors, there are therapeutic strategies that aim to enhance drug delivery to cancer cells by either decreasing the hyperpermeability of the blood vessels or by reversing the compression of blood vessels [46]. Lastly, our study suggests a new combination that renders CisPt more effective at lower doses but does not aim to solve the specificity problem of CisPt. The combination of mechanical stress and PBMCs together with CisPt has an increased effect as compared to CisPt being added alone to HepG2 cells.

### 4.4. Addressing Possible Limitations

First, the current investigation was only meant to be an initial feasibility study on using mechanical stress to destroy cancer cells in an* in vitro* culture. Obviously, an extensive series of future studies would need to be performed in animals and eventually humans before the clinical potential of mechanical stress for treating cancer could be realized. This was beyond the scope of the present study.

Second, only one type of cancer cell was used, namely, HepG2 cells. They were used because they have similar cell morphology as hepatocytes [20] and are frequently used to assess the immunomodulatory effect of immune cells on cancer cells [15]. However, the influence of mechanical stress with or without anticancer drugs and PBMCs on other types of cancer cells was beyond the scope of the current study.

Third, only one level (i.e., 300 rpm) and one type (i.e., horizontal circular motion) of mechanical stress were systematically evaluated. The effects of other levels (e.g., constant speed versus acceleration) and types (e.g., circular versus linear motion) of mechanical stress need to be examined to determine their influence on cancer cell death. Such factors would introduce different modes of stress application (e.g., compressive, tensile, or shear) to the cancer cells, which may or may not cause cancer cell death. For example, a prior* in vitro* model showed that moderate compressive stress enhanced cancer cell motility, whereas excessive compressive stress induced cancer cell death [39].

Fourth, the complexation/coupling between CisPt and carnosine was used as the only measure to assess the sequestration of CisPt in the cytoplasm. Although carnosine is known to chelate metal ions such as zinc, copper, vanadium [47], and platinum [18], the intracellular localization and trafficking of CisPt within the different cellular compartments need to be systematically followed. Examining the effect of mechanical stress on the complexation of CisPt with cytosolic ligands other than carnosine such as glutathione, ATP7B, and Atox1 needs to be studied to better understand the effect of mechanical stress at the molecular level [48].

Finally, for both cell viability and Caspase-3 expression, the sample size was slightly smaller than needed, respectively, yielding overall* post hoc* powers of 68% and 53% for all possible pairwise comparisons. However, for cell viability, 6 of 7 test groups compared to the control had an adequate sample size, that is, 78% to 100%. Moreover, Caspase-3 expression tests were only meant to act as supplemental indications of cell death, rather than relying on statistical analysis of the data for definitive conclusions.

## 5. Conclusions

This preliminary study proposes a novel method with potential application for cancer treatment. Mechanical stress + CisPt + PBMCs combination was the most effective combination in destroying cancer cells. Mechanical stress with or without CisPt or PBMCs alone was also effective. The usefulness of low doses of CisPt and PBMCs alone or combined is drawn into question. This is the first* in vitro* study on cancer cell death caused by mechanical stress, CisPt, and PBMCs. Most importantly, present results suggest that mechanical stress promotes the entry of CisPt to the nucleus, whether through freeing it from complexing with carnosine or through facilitating its transport to its pharmacological target site.
